# Relationships between pig farm management and facilities and lung lesions' scores and between lung lesions scores and carcass characteristics

**DOI:** 10.1186/s12917-024-03968-2

**Published:** 2024-03-28

**Authors:** Zbigniew Kuberka, John F. Mee, Aurelia Walaszek-Kayaoglu, Małgorzata D. Klimowicz-Bodys, Arkadiusz Dors, Anna Rząsa

**Affiliations:** 1 Private Practice, ul. Klonów 10A, Dobrzyca, 63-330 Poland; 2 Department of Immunology, Pathophysiology and Veterinary Preventive Medicine, Wroclaw University of Environmental and Life Sciences, ul. C. K. Norwida 31, Wroclaw, 50-375 Poland; 3 Animal and Bioscience Department, Teagasc the Agriculture and food Development Authority, Moorepark, Fermoy, Co. Cork Ireland; 4 Division of Infectious Diseases of Animals and Veterinary Administration, Department of Epizootiology and Clinic of Birds and Exotic Animals, Faculty of Veterinary Medicine, Wroclaw University of Environmental and Life Sciences, pl. Grunwaldzki 45, Wroclaw, 50-366 Poland; 5 Department of Preclinical Sciences and Infectious Diseases, Faculty of Veterinary Medicine and Animals Science, Poznań University of Life Sciences, ul. Wołyńska 35, Poznań, 60-637 Poland

**Keywords:** Pigs, Health monitoring, Lung scoring, Post-mortem examination, Farm questionnaire

## Abstract

**Background:**

The objective of this study was to examine the inter-relationships between pig farm management and facilities (as assessed by questionnaire) and post-mortem lung lesion (lung score assesment), which are the result of respiratory infections. The relationships between carcass characteristics and post-mortem lung lesion scores were also investigated.

**Results:**

Questionnaire responses were collected from 22 self-selecting pig farmers about their farm facilities/management and health condition of the respiratory system of pigs, including the occurrence of clinical respiratory signs, results of laboratory testing for respiratory pathogens, and the use of respiratory vaccines. When fatteners were sent to the abattoir, their carcasses (*n* = 1,976) were examined for evidence of respiratory disease by lung lesion (pleuritis pneumonia-like (PP-like) and enzootic pneumonia-like (EP-like) lesions) scoring and the *Actinobacillus pleuropneumoniae* Index (APPI) was calculated. Carcass characteristics were recorded and, retrospectively, the prevalence of cachectic pigs was calculated. Using these variables, the relationships between farm facilities/management and lung lesions scores and the relationships between the latter and carcass characteristics and cachexia were explored. The key findings relating farm facilities and management to lung lesions were: slatted floors were associated with significantly higher EP-like lesions scores than litter bedding in weaners, single-stage fattening in the same building was associated with significantly higher EP-like lesions scores than two-stage fattening, but herd size, stocking density, use of all-in/all-out (AIAO) rule, technological break duration and variation in daily temperature did not affect lung lesions scores. The key findings relating lung lesion scores to carcass characteristics were: a significant, negative correlation between EP-like scores and carcass weight but not with other carcass characteristics, a significant positive correlation between PP-like scores and carcass meat content and prevalence of cachectic carcasses and a significant positive correlation between lung APPI and prevalence of cachectic carcasses.

**Conclusions:**

It can be concluded that both farm facilities and management affect lung lesions scores and that the latter affect carcass characteristics. Lung lesion scoring is an inexpensive technique suitable for rapid monitoring of large numbers of carcasses that can be performed after animal slaughter. It provides useful information to inform producers about possible deficits in farm facilities or management and is a predictor of economic loss due to poorer quality carcasses.

**Supplementary Information:**

The online version contains supplementary material available at 10.1186/s12917-024-03968-2.

## Background

The intensification of pig production poses ongoing challenges for veterinarians in pig herd health management. High pig farm stocking rates provide favourable conditions for the transmission of many infectious pathogens [1, 2], which can lead to lower profitability of pig production.

The critical feature to maintaining a swine herd in health is a herd health programme including diagnosis of all diseases occurring in the herd, which include thorough anamnesis, clinical examinations and laboratory diagnostics as well as production data analysis. Nienhaus et al. [3] emphasised two groups of health indicators: resource-based and animal-based; both should be utilised, where data are available, to improve pig herd health [4].

One of the most cost-effective ways of gathering a comprehensive anamnesis is to use a standardized questionnaire [3, 5, 6]. In addition to anamnestic data, collection of pathological data, using scoring systems is valuable. Scoring methods are non-invasive, inexpensive and allow for an examination of a representative number of animals in a relatively short time [7–9].

Lung lesion scoring systems are now widely used internationally [9–11]. Lung scoring systems can detect the consequences of chronic infections, which are the most important from an economic point of view. The assessment is based primarily on the observation of the extent of lesions, mainly in the lung tissue and pleura. There are two common lesion types. The first type of lesion is caused most often by *Mycoplasma hyopneumoniae* (Mhp) [12], and is called enzootic pneumonia-like lesions (EP-like) or cranioventral pulmonary consolidation (CVPC) [7]. The second common lesion type is associated with inflammation of the lungs and pleura called pleuropneumoniae-like lesions (PP-like, pleuritis pneumonia). The pathogens causing these lesions are *Actinobacillus pleuropneumoniae* (App), *Haemophilus parasuis* (Hps), *Streptococcus suis* (Ss), *Mycoplasma hyorhinis* (Mhr), and less frequently, Mhp [13]. For a comprehensive assessment of pig herd health, anamnestic information needs to be combined with abattoir-collected data.

Hence, this study’s objective was to examine inter-relationships between farm management/facility data collected by questionnaire and pig health as assessed by abattoir lung scoring. In addition, the relationships between lung scoring and carcass characterises were also explored.

## Results

### Farm characteristics

#### Facilities and management

While the herd sizes varied between 50 and 1,400 sows, most herds had between 80 and 120 sows. The average number of animals per m^2^ for the whole room and in every pen in the fattening building was 1.19 and 1.21, respectively. On 10 (45.5%) farms, the fattening buildings had a concrete floor, 4 (18.2%) farms had both a concrete grate and a deep litter system, and 8 (36.4%) farms used a straw bedding system (5 deep and 3 shallow). The AIAO role was not followed in all production stages on any farm. This rule was used on 12 (54.4%) farms in the fattening stage. The majority of the surveyed farms (12; 54.4%) used one-stage fattening, while the remaining farms carried out two-stage fattening, and the fattening sectors were located several dozen to several hundred meters from the farrowing and nursing sectors. The technological break time in the fattening sector was from 0 to 17 days (average 4.3). However, in this sector, 2 (9.1%) farms did not apply the technological break.

#### Health status

**Fig. 1:**
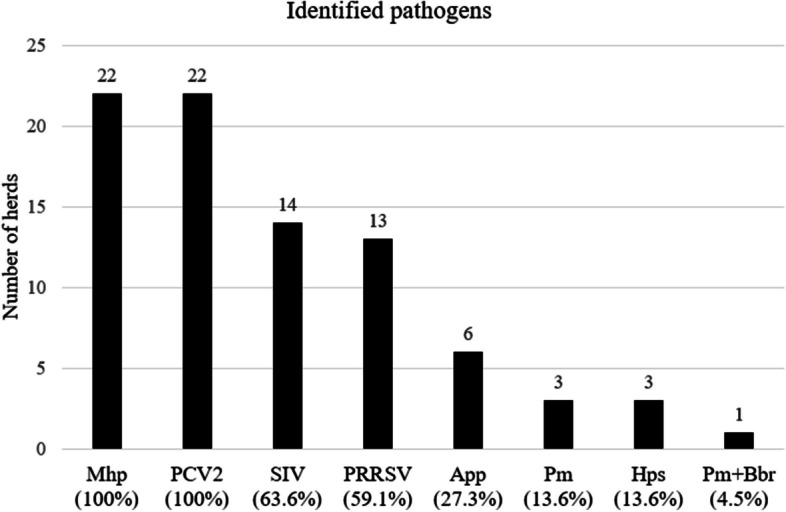
Percentage of 22 pig herds with laboratory-detected respiratory pathogens (based on questionnaire responses). Legend: Mhp (Mycoplasma hyopneumoniae); PCV2 (Porcine circovirus type 2); SIV (Swine influenza virus); PRRSV (Porcine reproductive and respiratory syndrome virus); App (Actinobacillus pleuropneumoniae); Pm (Pasteurella multocida); Hps (Haemophilus parasuis); Pm + Bbr (Pasteurella multocida + Bordetella bronchiseptica)

At least one infectious pathogen was detected on all farms within the last year by laboratory testing of pig samples (Fig. 1), varying between 3 and 8 pathogens per farm. The four farms where clinical symptoms were most often observed, were porcine reproductive and respiratory syndrome (PRRS) and Mhp positive, three of them additionally swine influenza (SI) positive, and two of them additionally App positive.

**Fig. 2:**
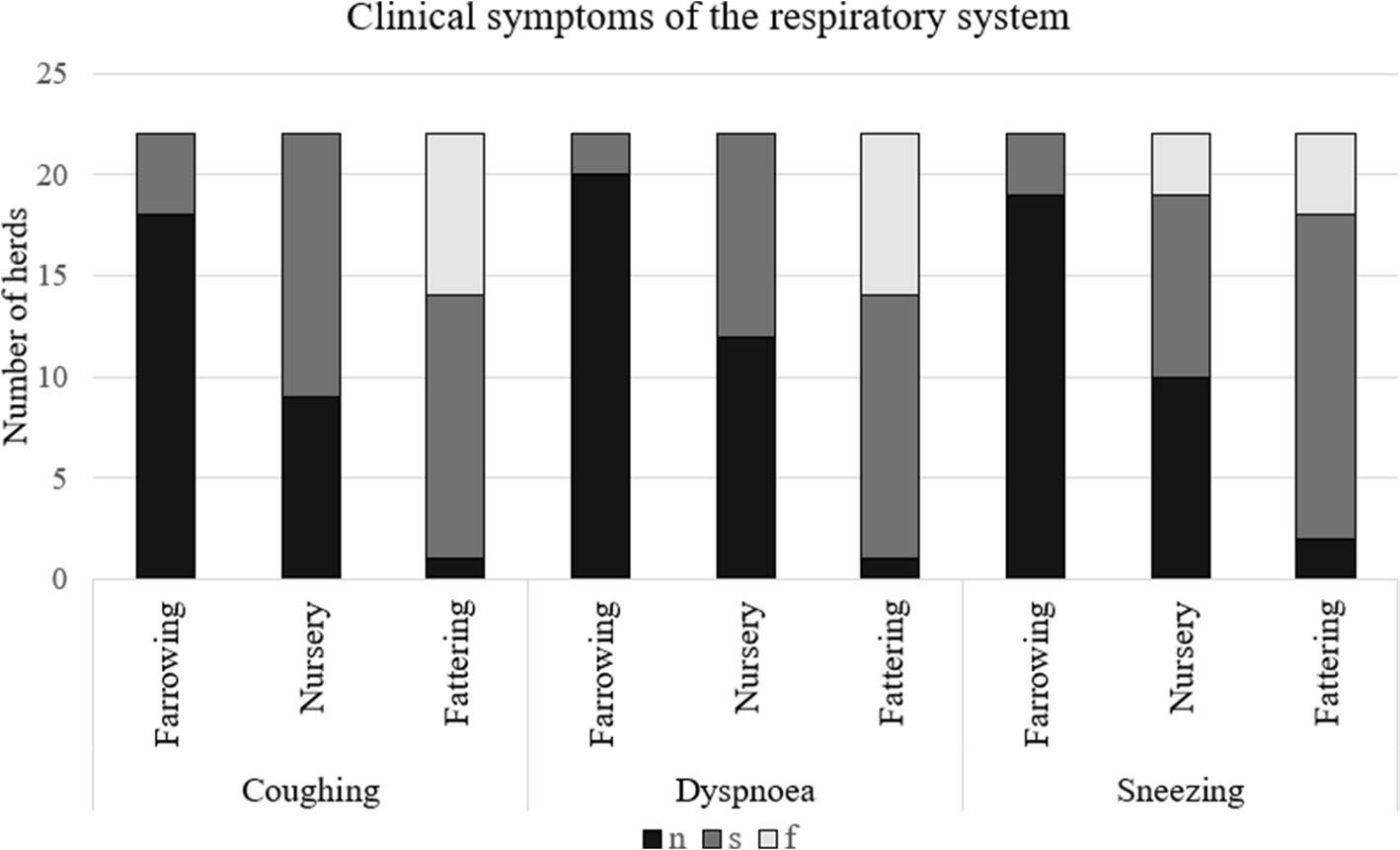
The occurrence of coughing, dyspnoea or sneezing in the farrowing, nursery or fattening stages. Legend: n – not observed; s – sporadic; f – frequent

 Clinical signs of respiratory disease (coughing, dyspnoea, sneezing) were reported in 21 (95.5%) farms. The occurrence and frequency of these signs in the different production stages (farrowing, nursing, and fattening) reported in the questionnaire by the owners of 22 farms are presented in Fig. 2.

#### Vaccination

All farmers vaccinated sows against erysipelas and parvoviruses. In 21 farms (95.5%), piglets were vaccinated against mycoplasmal pneumonia of swine (MPS; enzootic pneumonia, EP), and porcine circovirus associated diseases (PCVAD). Very few farmers vaccinated against pleuropneumonia (4.6%). None of the surveyed farms vaccinated piglets and only 10 (45.5%) farmers vaccinated sows against PRRS. These data are presented in Table 1.

**Table 1:** Vaccination against respiratory diseases in 22 pig herds based on questionnaire responses

Disease	Herds (%)
Mycoplasmal pneumonia of swine	95.5
Porcine circovirus disease	95.5
Atrophic Rhinitis	81.8
Swine Influenza	45.5
Porcine reproductive and respiratory syndrome	45.5
Porcine pleuropneumonia	4.6

### Lung characteristics

Of the 1,976 lungs of assessed fatteners, 22.6% had no pathological lesions (2.5 to 55%, by herd).

### EP-like lesions

EP-like lesions were present in all herds and were found in 1,452 (73.5%) of the lungs examined, varying between 37 and 94.7% by herd. Lesions of varying severity were observed as reflected in the point spread (1 to 21 points for individual lungs). The mean (SD) score for EP-like changes in all lungs was 3.38 ± 3.66 points. For lungs with lesions, the mean (SD) was 4.60 ± 3.57 points. Scars (fibrosis) were found in 11.6% of the examined lungs from 20 (90.9%) herds.

#### PP-like lesions

PP-like lesions were recorded in 12.1% of the examined lungs from 21 (95.5%) farms, varying between 2.4 and 43.2% in affected herds. The most frequent lesions were mild severity (0.24 ± 0.72 points). The mean (SD) score for all assessed lungs was 0,24 ± 0,72 and for lungs with PP-like lesions it was 1.96 ± 0.98 points. The mean *Actinobacillus pleuropneumoniae* Index (APPI) value was 0.19 and ranged from 0.2 to 0.89 in the herds where it was possible to calculate this index.

The frequency and severity of post-mortem lung lesions in pigs from 22 herds are presented in Table 2.

**Table 2:** The frequency and severity of post-mortem lung lesions (EP-like and PP-like) in pigs from 22 herds

Herd	Number of lungs assessed	Lungs without lesions % (n)	EP-like lesions (score 0–28)			PP-like lesions (score 1–4)			APPI
			Lungs % (n)	Average score (all lungs)	Average score (lungs with lesions)	Lungs % (n)	Average score (all lungs)	Average score (lungs with lesions)	
1	80	11.3 (9)	87.5 (70)	2.58	2.94	22.5 (18)	0.64	2.83	0.54
2	76	32.9 (25)	60.5 (46)	2.17	3.59	6.6 (5)	0.16	2.40	0.16
3	100	20.0 (20)	78.0 (78)	2.44	3.13	4.0 (4)	0.08	2.00	0.08
4	80	52.5 (42)	40.0 (32)	1.24	3.09	2.5 (2)	0.08	3.00	0.08
5	80	20.0 (16)	80.0 (64)	2.85	3.56	2.5 (2)	0.05	2.00	0.05
6	105	24.8 (26)	71.4 (75)	2.19	3.07	4.8 (5)	0.11	2.40	0.11
7	76	5.3 (4)	92.1 (70)	4.70	5.10	14.5 (11)	0.24	1.64	0.16
8	120	51.7 (62)	45.0 (54)	1.70	3.78	3.3 (4)	0.08	2.50	0.08
9	75	16.0 (12)	76.0 (57)	3.64	4.79	2.7 (2)	0.03	1.00	nd
10	86	47.7 (41)	47.7 (41)	0.90	1.88	3.5 (3)	0.03	1.00	nd
11	80	26.3 (21)	73.8 (59)	2.43	3.29	0 (0)	nd	nd	nd
12	120	10.8 (13)	88.3 (106)	5.68	6.43	16.7 (20)	0.24	1.45	0.15
13	75	5.3 (4)	94.7 (71)	6.08	6.42	18.7 (14)	0.28	1.50	0.16
14	80	23.8 (19)	70.0 (56)	2.75	3.93	11.3 (9)	0.21	1.89	0.18
15	75	41.3 (31)	49.3 (37)	1.25	2.54	6.7 (5)	0.09	1.40	0.05
16	102	8.8. (9)	88.2 (90)	4.62	5.23	18.6 (19)	0.39	2.11	0.33
17	140	4.3 (6)	92.9 (130)	5.47	5.89	17.9 (25)	0.31	1.72	0.21
18	75	25.3 (19)	70.7 (53)	4.67	6.60	80 (6)	0.16	2.00	0.13
19	81	4.9 (4)	84.0 (68)	3.22	3.84	43.2 (35)	0.98	2.26	0.89
20	100	55.0 (55)	37.0 (37)	0.70	1.89	5.0 (5)	0.06	1.20	0.02
21	90	6.7 (6)	92.2 (83)	4.98	5.40	28.9 (26)	0.56	1.92	0.44
22	80	2.5 (2)	93.8 (75)	7.22	7.69	18.8 (15)	0.36	1.93	0.26

Legend:* nd *No data

#### Carcass characteristics

The herd average carcass weight was 92.7 kg, varying between 81.2 and 103 kg by herd (Table 3). The herd average carcass lean meat content was 57.2%, varying between 53.1 and 61%, by herd. In total, 1,88% of carcasses were recorded as cachectic, varying between 0 and 4.9%, by farm.

**Table 3:** Selected parameters of fattening and slaughtering performance in individual herds

Herd	Carcass lean meat content (%)	Slaughter weight (kg)	CV of slaughter weight (%)	Cachexia frequency (%)
1	57.6	97.2	7.1	2.5
2	57.2	93.8	5.6	2.6
3	58.4	97.7	10.9	1.0
4	54.9	101.1	6.3	3.8
5	55.7	99.0	8.1	1.3
6	55.7	88.6	12.0	2.9
7	56.7	81.2	8.9	2.6
8	57.8	88.3	8.7	0.8
9	54.9	88.4	8.0	2.7
10	53.1	102.2	13.1	1.2
11	57.8	nd	nd	nd
12	57.5	86.2	12.8	1.7
13	56.5	91.9	10.3	2.7
14	nd	nd	nd	nd
15	61	88.1	11.4	0.0
16	59.9	85.2	9.2	2.0
17	57.2	103.5	7.0	3.6
18	57.9	87.5	9.4	0.0
19	60.1	93.3	9.8	4.9
20	57.7	93.2	8.5	0.0
21	56.7	93.2	5.7	2.2
22	55.8	84.8	9.0	2.5

Legend: *nd *No data

### Relationships between facilities/management and lung lesions

The relationships between all six facility/management variables and lung lesions scores were tested and statistically significant findings are reported. The size of the herd, use of the AIAO rule, the number of animals per 1 m^2^ of the building and the length of the technological break had no significant effect on the post-mortem lung assessment.

In the herds where the weaners were kept on the slatted floor, the mean EP-like lesions score was 3.64 (SD = 1.78), while in the herds where the animals were kept on litter, it was only 1.44 (SD = 0.67) (*p* = 0.035). In two-stage fattening farms, the average percentage of pigs with EP-like lesions was 60.8% which was lower (*p* = 0.04) than on farms where pigs were fattened in the same building (79.2%). However these results were not confirmed statistically.

### Correlations between lung lesion scores and carcass characteristics

EP-like lesion scores were negatively (rho = -0.08) correlated with carcass weight (*p* = 0.0015) but were not correlated with other carcass characteristics. PP-like lesion scores were positively (rho = 0.09) correlated with carcass lean meat content (*p* < 0,001). In addition, the mean PP-like lesion score was positively correlated (*r* = 0.59) with percentage of cachectic carcasses (*p* = 0.005). Moreover, there were positive correlations between the percentage of lungs with both type of lesions and APPI (*r* = 0.45, *p* = 0.04 and *r* = 0.60, *p* = 0.009, respectively) and the percentage of cachectic pigs.

## Discussion

### Facilities and management

The finding that floor type (slatted or semi-slatted vs. litter) is significantly associated with lung lesions confirms results from earlier studies [14–16]. The lower prevalence of lung lesions in pigs accommodated on litter systems is probably the result of better thermal comfort as well as improved welfare by reducing stress, which reduces susceptibility to respiratory disease [17, 18].

Respecting the AIAO rule important for animal health control, including respiratory diseases, and aims to limit the spread of pathogens between production sectors [19]. This rule was applied by only 54.5% of farms. The percentage of lungs showing pathological lesions was 14% higher in the herds where this rule was respected. This result is opposite to the expected one. However, adherence of the AIAO role was as reported by the owners and it could not be verified during the study. Farmers may claim to follow the AIAO rule, but often inadvertently fail to do so. Perhaps this rule was not fully implemented because of regrouping of some fatteners according to their body weight to obtain uniformity in slaughter weight [20, 21].

Two-stage fattening was associated with a significantly lower prevalence of EP-like lesions compared to fattening in one room. Similar results, although concerning PP-like lesions, were obtained in Italy [22]. The probable reason for the results found in the present study is lower antigenic pressure in the next building to which fatteners were moved. If animals stay in one building throughout the production cycle, they are exposed to increasing contamination of the environment with various pathogens until the end of the fattening period [23].

There was no association between the duration of the technological break and the severity of lung lesions, a result similar to that reported by Merialdi et al., [9]. However, a Spanish study found a positive correlation, indicating that adoption of a technological break can reduce the prevalence and severity of lesions in the lungs of fattening pigs [7]. Too short technological break time, and certainly its absence, may have an impact on the exacerbation of clinical symptoms and pathological lesions in the lungs observed during slaughterhouse audits. In many cases, such a situation is reflected in the lack of cleaning and disinfection or carrying out these activities in a careless manner, as well as non-compliance with the AIAO principle. Negligence in this area as risk factors for respiratory diseases in pigs was described by Fablet et al. [1].

### Health status and vaccination

There were no herds free from respiratory pathogens in this study and coinfections occurred commonly. The occurrence of coinfections may induce and/or intensify clinical symptoms and pathological lesions in the lungs [24, 25]. Moreover, the farms with the highest prevalence of respiratory clinical signs had the highest pig density in the fattening sector, which could induce the symptoms due to the easier horizontal transmission of pathogens [1, 26] and had a significant impact on the intensity and extent of lung lesions [19].

There was no metaphylactic use of antibiotics on the study farms. Antibiotics were used occasionally, to treat sick pigs, mainly during the nursery phase. A high adoption rate of vaccination against some respiratory diseases (MPS and PCVAD) was found in the examined herds (95.4%). In a previous Polish study the percentages of pig herds in which piglet vaccination against MPS and/or PCVAD was implemented was 63.5% and 49.7%, respectively [27]. This may reflect MPS and PCVAD status of the surveyed herds. In contrast, the farm-level adoption of pleuropneumonia vaccination (4.5%) was low and was similar to previously published data from Polish pig herds (6.1%) [27]. Analyses performed in 2015 showed that none of 140 farms surveyed used anti-influenza immunoprophylaxis [28], while in the current study, the percentage of farms using vaccination against swine influenza was 45.5%. In the present study 10 herds used PRRS vaccines for sows, (45.5%). Data from Germany [29] and Spain [7] show higher usage rates of these vaccines, 84% and 63.3%, respectively. In the current study, none of the surveyed herds used this method of immunoprophylaxis against piglets. In countries such as France and Belgium, the percentage of farmers using vaccination against PRRS in piglets was 7.2% [1] and 2% [8], respectively.

### Prevalence of EP-like lung lesions

EP-like lesions were detected in the lungs of fatteners from all herds, and in nearly 75% of all tested lungs. This prevalence is higher than that reported in all previous comparable studies internationally. For example, in the Balkan countries – 61% of lungs [30], in Italy – 59.6% [31], and in France – 69.3% [32]. However, while the prevalence of lungs with these lesions was high, the mean lesion score (3.34) was not dissimilar to those in previous reports; similar values were obtained in Spain [7], France [32, 33] and in Italy [30]. Apparent differences between studies could be explained by epidemiological factors such as prevalence of the underlying causal agents, differences in pig susceptibility, environmental factors such as climate, housing, or management features and methodological factors such as number of examined lungs or scoring methods [34].

The high prevalence of EP-like lesions (~ 75%) and scars (12%) in pigs, despite the use of respiratory vaccinations, is concerning. This may reflect the greater impact of facility and management factors in predisposing to respiratory disease than of vaccination to prevent respiratory disease. Other authors had similar observations and conclusions [35, 36]. Moreover, the lack of significant difference in the prevalence of EP-like lesions between herds vaccinated and non-vaccinated has also been described previously [9].

### Prevalence of PP-like lung lesions

This study showed a high prevalence of PP-like lesions at a herd-level (90%) and a lower prevalence at animal-level (11.9%). Similar herd-level (80%) and animal-level (from 11 to 12.5%) results have been reported in some studies internationally [37, 38]. However, in general, the herd-level prevalence reported here is much higher than comparable data internationally. For example, mean herd-level prevalence scores were 10% in Scotland [39], 20.8% in Belgium [8], 27% in Denmark [40], 26.8% in Spain [7], and 15% in France [31]. These discrepancies may result from different technologies in pig rearing in these countries, for example, respecting the AIAO rule (which was not respected in all investigated farms, in the this study). Also important for analysis is the method of data collecting about the herd. In present study, data were collected by the authors’ own observations and an interview with the owner, while in most other studies, these were only telephone interviews due to the large number of herds which the animals were assessed from.

The mean score for all examined lungs was 0.24, and the mean APPI value was 0.19. These findings have not been published for pig production systems in Poland before. Research conducted in Spain showed higher values, 0.5 and 0.4 [7], and in Italy 0.83 and 0.61, respectively [9]. The higher results from Spain and Italy may have been influenced by the fact that a large proportion of the tested lungs came from an area with a very high concentration of pig farms, accounting for 43% of the total pig production in Spain and 80% in Italy. According to the information available in the Local Data Bank of the Central Statistical Office of the Republic of Poland, in recent years the pig population in Wielkopolska averaged 32.45% (from 21.78 in 2012 to 36.7% in 2015) of the total pig production in Poland. The proportion of farms closed and open production cycles could also be influence this results [22], in our research they were the only farms with a closed production cycle. The higher results obtained in Italy may also be caused by the age of the slaughtered animals (9–10 months), while in Poland it was about 100 kg (5.5–6 months). The seroprevalence of the App in animals weighing 160 kg was higher than in lighter pigs [9]. The results of studies carried out in four Balkan countries showed comparable values for APPI (0.36) and the percentage of lungs with pleuritis (14.3%) [41]. In other European countries, similar values of 0.32 and 12.7%, respectively, were recorded [11].

### Relationships between lung lesions and carcass characteristics

The results reported here show that the prevalence, extent, and nature of lung lesions affect carcass characteristics, as previously documented [38, 42]. Specifically, the influence of EP-like lesions on carcass weight found here was previously reported in an English study [38]. The latter study demonstrated that higher scoring was associated with a reduction of 0.37 kg in carcass weight. In the same study, it was shown that the carcass weight of pigs with PP-like lesions in their lungs was, on average, 1.24 kg lighter than those with no adhesions. In agreement, in the present study, the mean score of PP-like lesions, APPI value, and the percentage of lung lesions were correlated with a higher percentage of cachectic carcasses. Additionally, the present results showed not only the negative impact of lesions, such as pleuritis, on carcass weight and prevalence of cachexia, but also a significant reduction in the quality of carcasses affected by adhesions in the thoracic cavity, rendering the carcasses less valuable. Ostanello et al. [31] demonstrated an adverse effect of EP-like lesions on carcass quality. They suggested that the observed decreasing of carcass quality may be related to metabolic disorders caused by hypoxia.

## Conclusions

Based on the findings in this study, it is concluded that EP-like lesions and PP-like lesions are frequently observed in slaughtered pigs in a Polish region with a high density of pig production, suggesting substantial economic losses. It is concluded that a risk assessment for respiratory disease should be routinely conducted using a structured questionnaire face-to-face interview between the veterinarian and the pig farmer as part of the farm herd health plan. The findings of this audit should be linked to post-mortem lung lesion scoring and the latter should be linked to carcass characteristics. This surveillance model provides the farmer and the veterinarian with data which can be used to drive decision-making on the farm to improve respiratory health. This model is convenient, inexpensive and can potentially improve pig farm health, welfare and profitability. The results of this study highlight that facility (type of floor) and management factors (number of fattening stages) were associated with the frequency and severity of EP-like lesions. Both investigated types of lesions were a risk factor for the presence of cachetic pigs. The high prevalence of pneumonic lesions justifies a need for future studies on lung lesions scoring in swine production in Poland.

### Methods

Data were collected from 22 self-selecting pig farmers about their farm facilities/management and pig respiratory health, including the occurrence of clinical respiratory signs, results of laboratory testing for respiratory pathogens, and use of respiratory vaccines. Additionally, when fatteners were sent to the abattoir, their carcasses were examined for evidence of respiratory disease by lung lesion scoring, carcass characteristics were recorded, and, retrospectively, the prevalence of cachectic pigs was calculated. Using these variables, the relationships between farm facilities/management and lung lesions scores and the relationships between the latter and carcass characteristics and cachexia were explored.

### Farms

This study was a part of a larger project investigating respiratory disease on pig farms in the western part of Poland (Wielkopolska), in a region with a high density of pig production [43, 44]. In total, 22 farrow-to-finish herds, varying in size from 50 to 1,400 sows, were used. These herds were chosen from the veterinary practitioner client database (*n* = 50 clients) of the first author. All herdowners were contacted by phone or personal communication and invited to enrol in a herd health study. Those that self-selected and who sold at least 75 fatteners in a single batch and did not administer antibiotics at least 14 days prior to slaughter were enrolled.

### Questionnaire

One veterinary practitioner (the first author) administered a questionnaire (*n* = 43 questions) to the 22 pig farm owners by on-farm, face-to-face interview. The questionnaire was designed, based on others available in the literature [12, 14, 16, 45]. The questionnaire had three sections (see Additional file 1). In this paper we address the relationships between the answers in the first section of the questionnaire: A - *Management and production organization* and lung lesions and carcass characteristics in the 12 months prior to the interview. We also describe the health status and vaccination regimes on the farms taken from section B (*Herd health status*) and C (*Veterinarian activity*).

#### Facilities/management variables

The following six variables were extracted from the questionnaire data to analyse the impact of facilities/management on the post-mortem lung assessment: herd size (no. sows), number of animals per 1 m^2^ of a building, floor type, AIAO rule, number of fattening stages, duration of the technological break.

### Lung lesion and cachexia scoring

Pigs from one batch of fatteners from each of the 22 herds were scored for lesions; in total, 1,976 pigs were scored; 75 to 140 per/herd. The lung scoring was carried out by the same veterinarian who administered the questionnaire – a swine disease specialist. Two types of lung lesions were scored: EP-like and PP-like.

The two-dimensional method described by Madec and Kobisch [46] was used to assess EP-like lesions, modified to account for the presence of scars which were defined as resolved lesions attributed to EP [40]. Lungs with no EP-like lesions were scored 0 and those with any such lesions 1.

PP-like lesions were scored using the Slaughterhouse Pleurisy Evaluating System (SPES) method [7, 9, 47, 48]. In relation to the group of carcasses, the APPI was calculated according to the formula:

$$\text{APPI}\text{ }\text{= }\frac{\text{number}\text{ }\text{of lungs with scores 2,3,4}}{\text{total number of lungs examined}}\text{ }\text{*}\text{ }\text{average score attributable A.P.}$$

where:

score 2 – limited inflammatory lesions with slight to moderate extensions into one of the diaphragmatic lobes;

score 3 – lesions like 2, but bilateral, in one of the diaphragmatic lobes can be extensive;

score 4 – severely extended lesions, at least 1/3 of both diaphragmatic lobes.

A.P. – average lungs’ punctation for lungs with 2,3,4 score.

Animals were retrospectively classified as cachectic if their carcass weight was less than two standard deviations below the mean of the batch.

### Carcass characterises

The carcass weight and lean meat content were recorded using EU-approved equipment in the slaughterhouses.

### Statistical analysis

The relationships between the six facilities/management variables in the questionnaire and the frequency and severity of lung scores and cachectic pigs were explored and the inter-relationships between these outcome variables were also examined. The continuous variables were subjected to the W. Shapiro-Wilk test for normality and based on the distribution outcome, the Pearson or Rho-Spearman test was chosen. Specifically, the Pearson test was used to determine the relationships between the lung lesion score (EP- or PP-like) and percentage of lungs with lesions and average carcass weight, lean meat content, and percentage of cachectic pigs in a given herd batch. Additionally, the Rho-Spearman correlations (rho) were calculated for individual fatteners between lung scoring evaluations, carcass weight and lean meat content. Data are presented as mean and standard deviation SD (±), number (n), percentage (%) and coefficient of variability – CV (%). The significance level adopted in all tests was *p* < 0.05. Statistica 8.0 and Microsoft Excel with the Real Statistics Resource Pack for Excel 2013/2016 were used to analyse the data.

### Supplementary Information

**Supplementary Material 1.**

## Data Availability

The datasets used and analysed during the current study are included in the article. More details of the information of the cases are not available for public access because of privacy concerns, but available from the corresponding author on reasonable request.

## Abbreviations

PP: Like–pleuritis pneumonia like
EP: Like–enzootic pneumonia like
APPI: *Actinobacillus pleuropneumoniae* Index
AIAO: All–in/all–out
Mhp: *Mycoplasma hyopneumoniae*
CVPC: Cranioventral pulmonary consolidation
App: *Actinobacillus pleuropneumoniae*
Hps: *Haemophilus parasuis*
Ss: *Streptococcus suis*
Mhr: *Mycoplasma hyorhinis*
PRRS: Porcine reproductive and respiratory syndrome
MPS: Mycoplasmal pneumonia of swine
EP: Enzootic pneumonia
PCVAD: Porcine circovirus associated disease
SPES: Slaughterhouse Pleurisy Evaluating System
