# Supplementary material for: Relationships between pig farm management and facilities and lung lesions' scores and between lung lesions scores and carcass characteristics
Source: BMC Vet Res. 2024 Mar 28;20:124. doi: 10.1186/s12917-024-03968-2 (PMC10976837; doi:10.1186/s12917-024-03968-2)
Supplement: Supplementary file 1 — Supplementary Material 1. [file 12917_2024_3968_MOESM1_ESM.docx]

**Additional file 1.** Herd questionnaire

| **Section A - *Management and production organization*** | |
| --- | --- |
| 1. Herd number | |
| 1. Number of sows in the basic herd | |
| 1. Number of gilts | |
| 1. Number of boars | |
| 1. Production rhythm (1-, 3-, 4- weeks, none) | |
| 1. Nutrition system | |
| 1. Number of rooms and pens in the farrowing sector | |
| 1. Number of rooms and pens in the nursery | |
| 1. Number of pigs in one nursing room and the nursing area | |
| 1. Number of pigs in one pen in the nursing room and the area of the pen | |
| 1. Number of rooms and pens of in the fattening sector | |
| 1. Number of pigs in one fattening room and the fattening area | |
| 1. Number of pigs in one pen in the fattening building and the area of the pen | |
| 1. Genetics | |
| 1. Fostering | |
| 1. Age of weaning | |
| 1. Room heating (what kind, age of the animals) | |
| 1. Body weight at weaning | |
| 1. Number of fattening stages | |
| 1. Separate buildings for every stage (how many for each stage) | |
| 1. Fattening at other place | |
| 1. Age and body weight when moving to the next stage of fattening | |
| 1. Respect of AIAO at: | |
| 1. farrowing sector | |
| 1. nursery | |
| c) fattening sector | |
| 24. Quarantine | |
| 25. Floor type (litter, grate, solid): | |
| 1. farrowing sector | |
| 1. nursery | |
| 1. fattening sector | |
| 26. Ventilation type: | |
| 1. gravity | |
| 1. mechanical | |
| 27. The length of the technological break: | |
| 1. farrowing sector | |
| 1. nursery | |
| 1. fattening sector | |
| 28. Temperature + daily amplitude: | |
| 1. farrowing sector | |
| 1. nursery | |
| 1. fattening sector | |
| 29. Morbidity: | |
| 1. age | |
| 1. percentage | |
| 1. treatment (what drugs, length and route of administration, percentage of animals) | |
| 30. Mortality | |
| 31. Reproductive performance: | |
| 1. total number of piglets born / sow / year | |
| 1. number of piglets born alive / sow / year | |
| 1. number of weaned piglets / sow / year | |
| 1. number of piglets sold / sow / year | |
| 1. number of litters / sow / year | |
| 1. insemination efficiency | |
| 32. Loses: | |
| 1. farrowing sector | |
| 1. nursery | |
| 1. fattening sector | |
| 33. Duration of fattening | |
| 34. Body weight of the fattening pigs sold | |
| 35. Herd replacement (%) | |
| **Section B - Herd health status** | |
| 1. Based on laboratory tests: |  |
| 1. Porcine Reproductive and Respiratory Syndrome Viruses (Porcine Arterivirus) | |
| 1. *Mycoplasma hyopneumoniae* | |
| 1. Porcine circovirus type 2 | |
| 1. *Actinobacillus pleuropneumoniae* | |
| 1. Swine Influenza Viruses | |
| 1. *Streptococcus suis* | |
| 1. *Haemophilus parasuis* | |
| 1. *Erysipelothrix rhusiopathiae* | |
| 1. *Pasteurella multocida* | |
| 1. Symptoms from the respiratory tract: | |
| 1. cough (age, symptom severity: common / sporadic) | |
| i) farrowing sector | |
| ii) nursery | |
| iii) fattening sector | |
| 1. dyspnoea (age, symptom severity: common / sporadic) | |
| i) farrowing sector | |
| ii) nursery | |
| iii) fattening sector | |
| 1. sneezing (age, symptom severity: common / sporadic) | |
| i) farrowing sector | |
| ii) nursery | |
| iii) fattening sector | |
| 1. Number of coughing attacks through 2-3 min. (in one room or neighbor pens) / number of pigs: | |
| 1. farrowing sector | |
| 1. nursery | |
| 1. fattening building | |
| 1. Number of pigs coughing through 2-3 min. (in one room or neighbour pens) / number of pigs | |
| 1. farrowing sector | |
| 1. nursery | |
| 1. fattening sector | |
| **Section C - Veterinarian activity** | |
| 1. Vaccination (product, age of animals during administration, doses) |  |
| 1. Porcine Reproductive and Respiratory Syndrome (PRRS) | |
| 1. Enzootic pneumonia | |
| 1. Porcine circovirus-associated diseases (PCVAD) | |
| 1. Pleuropneumonia | |
| 1. Swine influenza (SI) | |
| 1. Atrophic rhinitis | |
| 1. Erysipelas | |
| 1. Glässer’s Disease | |
| 1. Others: | |
| 1. Metaphylactic treatment: | |
| i) a type of antibiotic | |
| ii) start of administration (age) | |
| iii) end of administration (age) | |
| iv) route of administration | |
| 1. iron supplementation (age, how many times) | |
| 1. castration (age) | |
| 1. tail clipping (age) | |
| 1. tooth shortening (age) | |
| 1. Sanitization: | |
| 1. farrowing sector | |
| 1. nursery | |
| 1. fattening sector | |
| 1. Disinfection: | |
| 1. farrowing sector | |
| 1. nursery | |
| 1. fattening sector | |
